# Visual lung ultrasound protocol (VLUP) in acute respiratory failure: description and application in clinical cases

**DOI:** 10.1007/s10877-024-01144-3

**Published:** 2024-03-09

**Authors:** A. Bianchini, Irene Sbaraini Zernini, G. Notini, E. Zangheri, C. Felicani, G. Vitale, A. Siniscalchi

**Affiliations:** 1 Postoperative and Abdominal Organ Transplant Intensive Care Unit, IRCCS Azienda Ospedaliero-Universitaria di Bologna, Bologna, 40138 Italy; 2 Department of Medical and Surgical Sciences (DIMEC), University of Bologna, Bologna, 40126 Italy; 3 Anesthesia and Pain Therapy Unit, IRCCS Azienda Ospedaliero-Universitaria di Bologna, Bologna, 40138 Italy; 4 UOC Medicina ad Indirizzo Metabolico Nutrizionale. Policlinico di Modena, AOU Modena, Via del Pozzo, 71, Modena, Italy; 5 Internal Medicine Unit for the Treatment of Severe Organ Failure, IRCCS Azienda Ospedaliero- Universitaria di Bologna, Bologna, 40138 Italy

**Keywords:** Lung ultrasound, Point-of-care ultrasound, Visual lung ultrasound protocol (VLUP), Acute respiratory failure, Lung ultrasound score

## Abstract

Lung ultrasound (LUS) is widely used as a diagnostic and monitoring tool in critically ill patients. Lung ultrasound score (LUSS) based on the examination of twelve thoracic regions has been extensively validated for pulmonary assessment. However, it has revealed significant limitations: when applied to heterogeneous lung diseases with intermediate LUSS pattern (LUSS 1 and 2), for instance, intra-observer consistency is relatively low. In addition, LUSS is time-consuming and a more rapid overview of the extent of lung pathology and residual lung aeration is often required, especially in emergency setting. We propose a Visual Lung Ultrasound Protocol (VLUP) as a rapid monitoring tool for patients with acute respiratory failure. It consists of a probe sliding along the mid-clavicular, mid-axillary and scapular lines in transversal scan. VLUP allows a visualization of a large portion of the antero-lateral and/or posterior pleural surface. Serial assessments of two clinical cases are recorded and visually compared, enabling rapid understanding of lung damage and its evolution over time. VLUP allows a semi-quantitative and qualitative point-of-care assessment of lung injury. Through this standardized approach it is possible to accurately compare subsequent scans and to monitor the evolution of regional parenchymal damage. VLUP enables a quick estimation of the quantitative-LUSS (qLUSS) as the percentage of pleura occupied by artifacts, more suitable than LUSS in inhomogeneous diseases. VLUP is designed as a standardized, point-of-care lung aeration assessment and monitoring tool. The purpose of the paper is to illustrate this new technique and to describe its applications.

**Supplementary Information:**

The online version contains supplementary material available at 10.1007/s10877-024-01144-3.

## Introduction

Bedside lung ultrasound (LUS) is widely used as a diagnostic and monitoring tool in critically ill patients [1, 2]. LUS score (LUSS), based on examination of twelve chest areas, has been extensively validated for lung aeration assessment but has shown essential limitations. For instance, when used to quantify heterogeneously distributed lung diseases such as acute respiratory distress syndrome (ARDS), pulmonary contusion or ventilator-associated pneumonia (VAP), LUSS can generate different scores due to the high dependency of the operator in evaluating the intermediate LUS scores pattern (LUSS 1 and 2) [3, 4]. Some authors also suggest that the coalescence of B lines (LUSS score 2) is inappropriate, as it overestimates pulmonary aeration loss [5–7]. Furthermore, in inhomogeneous lung pathology, the intercostal space chosen within the same thoracic area and the score assigned when different pathological patterns coexist can lead to high-scoring variability, affecting the results’ reproducibility.

A non-standardized approach (e.g. different intercostal spaces analyzed from the same thoracic area), the presence of confounding factors (e.g. different imaging parameters and types of probe) and the subjectivity of the analysis can increase inter- and intra-observer variability [8].

Finally, LUSS is time-consuming and not strictly suitable in emergency contexts or in uncooperative patients.

## Aims

**Fig. 1:**
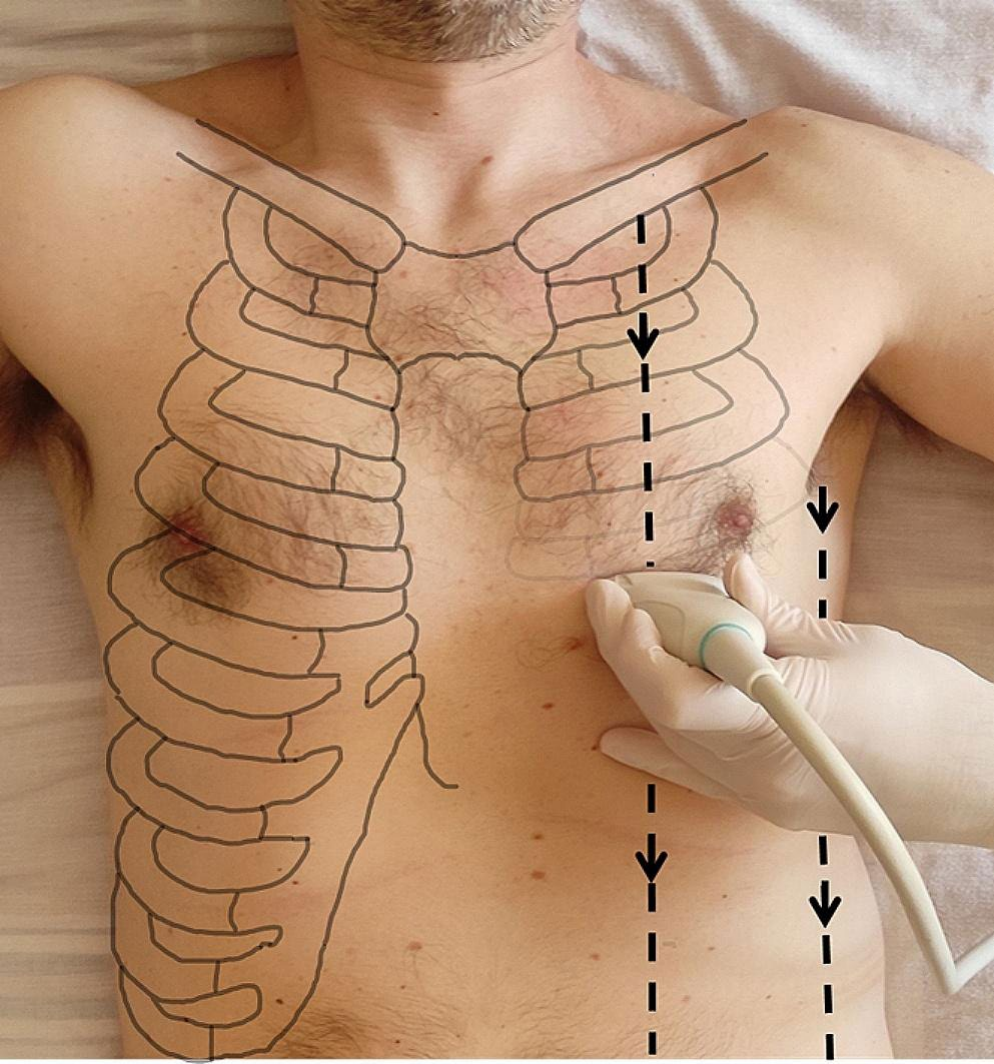
VLUP standardized transversal approach. The continuous sliding transversal scan is performed from the first intercostal space to the last viewable, along the midclavicular and the mid-axillary lines. The scapular line is used as a scan guide in evaluating the posterior chest wall

Considering the need for a standardized, quick, and easily repeatable lung evaluation tool, we propose a Visual Lung Ultrasound Protocol (VLUP). It consists of a continuous transverse scan along all explorable intercostal spaces of the anterior, lateral and posterior thoracic region (Fig. 1; Video 1).

We designed VLUP as a lung point-of-care ultrasound (POCUS) assessment. So, we described two exemplary clinical cases to test the utility of the VLUP protocol to monitor pulmonary pathology and solve significant patient management issues quickly.

## Methods

### Ultrasound technique

VLUP evaluates a wide pleural surface using an intercostal ultrasound scan with a standardized transversal approach. The linear probe is slid on the thoracic wall from one intercostal space to the other, in anterior, lateral and posterior thoracic regions. The continuous sliding transversal scan is performed from the first intercostal space to the last viewable, along the mid-clavicular and the mid-axillary lines. (Fig. 1) The scapular line is used as a scan guide in evaluating the posterior chest wall.

Each intercostal space is displayed for 1–2 s at least. A video clip is then recorded for each patient noting the date and region scanned [Video 1]. Comparative visualization of the clips performed on different days allows a quick evaluation of the pulmonary pathology development [Video 2].

We suggest to use a linear probe in normal weight patients and a convex probe in obese ones, due to its greater penetration capacity. The depth must be set greater than twice the depth of the pleura in order to see the first A-line and quickly identify airy regions.

### Technical application

We have experienced the utility of the VLUP protocol in monitoring various pulmonary pathologies, such as ARDS, VAP, pulmonary edema and pulmonary contusion, both in adult and child patients.

In addition, we have verified the correlation with other diagnostic imaging tools, primarily computed tomography (CT) and radiography [Video 1].

The following describes VLUP application in two clinical cases of acute respiratory failure due to bilateral pneumonia in patients admitted to our Intensive Care Unit (ICU) at IRCCS S. Orsola-Malpighi Hospital, Bologna, in 2023.

This study was conducted following ethical guidelines of the World Medical Association’s Declaration of Helsinki and guidelines for Good Clinical Practice. Informed consent from each patient was obtained. Finally, we have attached more explanatory ultrasound images and videos of the two cases, performed and then compared by a single trained physician, with expertise on LUS. We use a General Electric LOGIQ S8 ultrasound machine with a L3-12-D linear probe to realize US exams.

### Cases’ description

#### Case 1 [Video 2

A 57-year-old woman was hospitalized for acute on chronic liver failure secondary to alcoholic disease; there were no other diseases in the medical history. The hospitalization was complicated by a bilateral bacterial pneumonia requiring mechanical ventilation; the CT scan showed diffuse ground-glass opacities, while the bronchial wash culture resulted positive for extended-spectrum beta-lactamase Escherichia Coli, so the patient began antimicrobial therapy with ertapenem. The first VLUP evaluation (day 1) was characterized by diffuse B-profile and bilateral confluent subpleural consolidations. The second VLUP assessment (day 2) showed the extent of right apical consolidations ( C-profile) despite a positive end-expiratory pressure (PEEP) > 10 cmH2O. We then performed guided LUS recruitment maneuvers, gradually increasing the insufflation pressure until the airway opening pressure was reached [Video 3] and increasing the PEEP following the ultrasound protocol proposed by Tusman et al. [9].

In this case, the day 1 and day 2 LUS scores remained unchanged (LUSS 24) due to a slight improvement in left lung ventilation. Considering alone, LUSS value underestimated the worsening of pulmonary regional aeration and the need for recruitment maneuvers. Conversely, apical consolidation was quickly identified by visually comparing the VLUP of day 1 with that of day 2 [Video 2], suggesting optimization of ventilation pressures.

The patient’s clinical conditions improved after the treatment with the targeted antibiotic therapy, with progressive clinical improvement and weaning from mechanical ventilation within five days, so the ICU discharged the patient.

#### Case 2 [Video 4

A 62-year-old man was hospitalized for aspiration pneumonia, with a predominantly perihilar involvement, as shown in the CT scan. The patient suffered from a liver alcoholic cirrhosis complicated with oesophageal varices, ascites and recurrent encephalopathy.

**Fig. 2:**
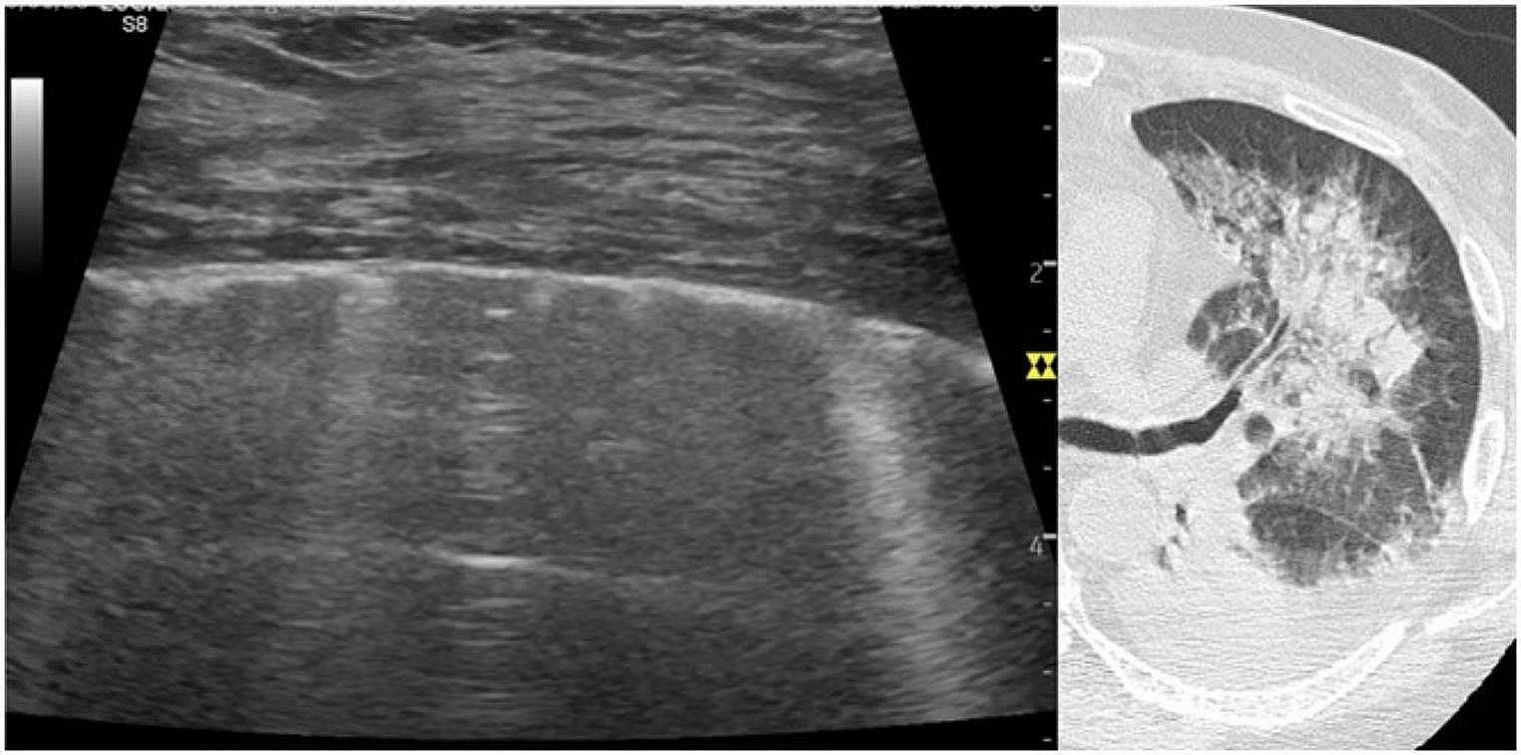
Transverse lung ultrasound approach in aspiration pneumonia with perihilar involvement. The scan was performed along the mid-axillary line (video 2 shows VLUP anterior scans from the same patient). Transverse ultrasound scan allows for visualization of a larger pleural surface without rib interruption. The pleura is only partially involved and ultrasound underestimates the damage. VLUP showed separate B-lines and small areas of confluent B-lines, without any consolidation (non-homogeneous ultrasound pattern)

Aspiration pneumonia is a condition classically underestimated by LUS as the pathology does not reach the pleura. LUS showed separate B-lines and small areas of confluent B-lines ( B-profile), without any consolidation (Fig. 2; Video 4). Due to the non-homogeneous ultrasound pattern, LUSS assumed different values according to the operator who performed the exam (range from 8 to 14).

In this case, VLUP provided a more complete and panoramic view of inhomogeneous lung disease and allowed us to monitor the pneumonia evolution accurately by visualizing the comparative extension of pleural injury. A chest CT scan confirmed the bilateral interstitial pneumonia with diffuse consolidations and pleural effusions.

The patient required high flow nasal cannula and non-invasive ventilation (NIV); the bronchial wash culture resulted positive for Escherichia Coli, Klebsiella Pneumoniae, and Enterobacter Cloacae. He was treated with steroids and targeted antimicrobial therapy with piperacillin-tazobactam, linezolid and prophylactic anidulafungin for 6 days, until complete recovery.

## Discussion

LUS is recommended to assess the severity and progression of lung disease over time [1, 2]. The most validated quantitative evaluation tool is LUSS, which evaluate the regional aeration of twelve thoracic areas, graded between 0 and 3 [1]. Nonetheless, it has demonstrated some limits over time and several modified scores have been proposed [7, 9]. Case 2 shows how LUSS can generate variable scores if different pathological ultrasound patterns coexist in the same chest area or the same ultrasound scan, as in inhomogeneous lung disease (Video 6, Video 7). Furthermore, LUSS images are often not directly compared due to the difficulty of memorizing and archiving the twelve images or to the limited time available, especially in emergencies. In these cases, the total LUSS value is used alone (without images), although it has a limited role in monitoring the regional evolution of lung damage, as shown in case 1.

The requirement for a rapid POCUS lung evaluation has grown and it has been recently recommended for pleural effusion, pneumonia, and interstitial syndrome diagnosis [10, 11]. We proposed VLUP as a qualitative, semi-quantitative, quick and repeatable lung aeration assessment and monitoring tool.

VLUP is based on intercostal ultrasound scanning with a transverse standardized approach. The transverse ultrasound scan allows visualization of a larger pleural surface than the longitudinal one which is limited by the width of the intercostal space (Fig. 2; Video 5). The transversal scan provides a more panoramic view of the pulmonary disease and the extent of lung damage [2, 5–7]. The continuous sliding transversal scan from one intercostal space to the other expands the panoramic assessment to the entire chest wall.

Furthermore, the standardization of the sliding technique along the anatomical lines (mid-clavicular, mid-axillary and scapular lines) allows us to compare subsequent scans accurately and monitor the evolution of regional parenchymal damage. Recording a video clip of the standardized continuous ultrasound scan facilitates visual comparison of the daily evolution of lung disease and pulmonary aeration [Video 2]. A semi-quantitative and qualitative visual assessment is therefore easily performed. It is possible to estimate the degree of variation in pulmonary aeration (increased, reduced, unchanged) based on the extent of lung damage and the type of ultrasound pattern ( A-profile > B-profile > C-profile).

In addition to longitudinal monitoring of lung disease, we also used VLUP to evaluate the patient with respiratory failure. Identifying A, B or C profiles allowed us to understand the cause of respiratory failure quickly and guide targeted therapies and procedures such as thoracentesis, fiberoptic bronchoscopy or diuretic therapy [12–14]. VLUP enables a quick estimation of the qLUSS as the percentage of pleura occupied by artifacts [7, 9]. qLUSS greater than 2/3 of the entire parenchyma (qLUSS ≥ 5/6) can predict NIV failure and the need for orotracheal intubation [9]. In this context, VLUP guided the patient’s admission to a ward with adequate monitoring and ventilatory support. Finally, VLUP allowed us to rapidly detect the presence of non-ventilated lung areas, recruitable by positive pressure (as shown by case 1, video 3) or prone position [13, 15]. In some cases, VLUP has suggested the presence of hyperinflated areas, characterized by many A lines and a reduced pleural sliding [16], leading to more protective ventilation [Video 5].

VLUP required a continuous scan of the anterior, lateral and posterior thoracic region, to provide a complete lung aeration assessment. Otherwise, considering known and ICU patients, even hard to mobilize as those described above, it is possible to reduce monitoring to the anterior and lateral regions only, aware of the limits of excluding the posterior scan.

Inter-operator variability was minimized by using the same sonographer for acquisition and comparative evaluations.

A VLUP limit is not to use a numerical quantification, which could be exceeded by automation and computer-assisted algorithms [3, 5]. The use of automation and computer-assisted algorithms could reduce inter-operator variability by aligning different settings and by standardizing the quantification of lung pathology through computerized image analysis. Its potential implementation in lung ultrasound may be a valuable future resource capable of overcoming many limitations and improving patient care [17]. The VLUP systematic, quick and well-defined approach could be helpful in further studies regarding a computer analysis of lung damage through artificial intelligence.

## Conclusion

VLUP is a new approach to LUS for a POCUS evaluation and monitoring of pulmonary disease. Its standardized approach allows a semi-quantitative and qualitative assessment of lung injury. Estimating qLUSS, namely the percentage of pleura occupied by artifacts, may overcome the limits of LUSS in evaluating inhomogeneous lung diseases. Moreover, it permits to compare accurately subsequent scans and to monitor the evolution of regional parenchymal damage. VLUP allows to overcome some limitations of LUSS and can become a useful tool for developing a computerized analysis of lung damage.

Further prospective studies are needed to assess its impact on diagnosing and managing patients with acute respiratory failure, particularly in specific populations and in different contexts.

### Electronic supplementary material

Below is the link to the electronic supplementary material.

Supplementary Material 1: Video 1. VLUP in patient with ARDS – mid-clavicular view. The continuous sliding transversal scan is performed along the right mid-clavicular lines (right anterior thoracic regions). VLUP demonstrates diffuse coalescent B-profile and confluent consolidations. The severity of lung involvement is similar to CT.

Supplementary Material 2: Video 2. VLUP evaluation in patient with ARDS. VLUP performed in the same anterior scans (along the right mid-clavicular line) on two successive days (day 1 and day 2). VLUP clearly showed the extent of apex consolidations.

Supplementary Material 3: Video 3. LUS recruitment maneuvers. When the airway opening pressure is reached, there is an improvement in pulmonary aeration with a clear reduction in consolidated areas.

Supplementary Material 4: Video 4. VLUP in aspiration pneumonia with perihilar involvement. The scan was performed along the bilateral mid-clavicular line (Fig. 2 shows VLUP lateral scans from the same patient). The pleura is only partially involved and ultrasound underestimates the damage. VLUP showed separate B-lines and small areas of confluent B-lines, without any consolidation (non-homogeneous ultrasound pattern).

Supplementary Material 5: Video 5. Area of suspected hyperinflation in a patient with ARDS (A-lines > 5 and decreased pleural sliding).

Supplementary Material 6: Video 6. Pneumonia with a non-homogeneous ultrasound pattern: in the same thoracic area there may be different LUSSs in different intercostal spaces.

Supplementary Material 7: Video 7. Pneumonia with a non-homogeneous ultrasound pattern: in the same thoracic area there may be different LUSSs in different intercostal spaces.

## Abbreviations

CT: Computed Tomography
ICU: Intensive Care Unit
LUS: Lung Ultrasound
LUSS: Lung Ultrasound Score
NIV: Non-Invasive Ventilation
qLUSS: quantitative-Lung Ultrasound Score
VLUP: Visual Lung Ultrasound Protocol
PEEP: Positive End-Expiratory Pressure
POCUS: Point-of-care Ultrasound
ARDS: Acute Respiratory Distress Syndrome
VAP: Ventilator-Associated Pneumonia

## References

[1] Volpicelli G, Elbarbary M, Blaivas M, Lichtenstein DA, Mathis G, Kirkpatrick AW, International Liaison Committee on Lung Ultrasound (ILC-LUS) for International Consensus Conference on Lung Ultrasound (ICC-LUS) (2012). International evidence-based recommendations for point-of-care lung ultrasound. Intensive Care Med.

[2] Demi L, Wolfram F, Klersy C, De Silvestri A, Ferretti VV, Muller M, Miller D, Feletti F, Wełnicki M, Buda N, Skoczylas A, Pomiecko A, Damjanovic D, Olszewski R, Kirkpatrick AW, Breitkreutz R, Mathis G, Soldati G, Smargiassi A, Inchingolo R, Perrone T. New International Guidelines and Consensus on the Use of Lung Ultrasound. J Ultrasound Med., Mento F, Khan U, Faita F, Smargiassi A, Inchingolo R, Perrone T, Demi L. State of the Art in Lung Ultrasound, Shifting from Qualitative to Quantitative Analyses. Ultrasound Med Biol. 2022;48(12):2398–2416. 10.1016/j.ultrasmedbio.2022.07.007. Epub 2022 Sep 23.

[3] Lerchbaumer MH, Lauryn JH, Bachmann U, Enghard P, Fischer T, Grune J, Hegemann N, Khadzhynov D, Kruse JM, Lehner LJ, Lindner T, Oezkan T, Zickler D, Kuebler WM, Hamm B, Eckardt KU, Muench F (2021). Point-of-care lung ultrasound in COVID-19 patients: inter- and intra-observer agreement in a prospective observational study. Sci Rep.

[4] Mongodi S, De Luca D, Colombo A, Stella A, Santangelo E, Corradi F, Gargani L, Rovida S, Volpicelli G, Bouhemad B, Mojoli F (2021). Quantitative lung Ultrasound: technical aspects and clinical applications. Anesthesiology.

[5] Mojoli F, Bouhemad B, Mongodi S, Lichtenstein D (2019). Lung ultrasound for critically ill patients. Am J Respir Crit Care Med.

[6] Mongodi S, Bouhemad B, Orlando A, Stella A, Tavazzi G, Via G, Iotti GA, Braschi A, Mojoli F (2017). Modified lung ultrasound score for assessing and Monitoring Pulmonary Aeration. Ultraschall Med.

[7] Soldati G, Smargiassi A, Inchingolo R, Buonsenso D, Perrone T, Briganti DF, Perlini S, Torri E, Mariani A, Mossolani EE, Tursi F, Mento F, Demi L (2020). Proposal for international standardization of the use of lung ultrasound for patients with COVID-19. J Ultrasound Med.

[8] Tusman G, Acosta CM, Costantini M (2016). Ultrasonography for the assessment of lung recruitment maneuvers. Crit Ultrasound J.

[9] Biasucci DG, Buonsenso D, Piano A, Bonadia N, Vargas J, Settanni D, Bocci MG, Grieco DL, Carnicelli A, Scoppettuolo G, Eleuteri D, DE Pascale G, Pennisi MA, Franceschi F, Antonelli M (2021). Gemelli Against COVID-19 Group. Lung ultrasound predicts non-invasive ventilation outcome in COVID-19 acute respiratory failure: a pilot study. Minerva Anestesiol.

[10] Jarman RD, McDermott C, Jenssen C, Sidhu PS (2023). EFSUMB Clinical Practice guidelines for Point-of-care Ultrasound: Part one (Common Heart and Pulmonary Applications) LONG VERSION. Ultraschall Med.

[11] Hussain A, Via G, Melniker L, Goffi A, Tavazzi G, Neri L, Villen T, Hoppmann R, Mojoli F, Noble V, Zieleskiewicz L, Blanco P, Ma IWY, Wahab MA, Alsaawi A, Al Salamah M, Balik M, Barca D, Bendjelid K, Bouhemad B, Bravo-Figueroa P, Breitkreutz R, Calderon J, Connolly J, Copetti R, Corradi F, Dean AJ, Denault A, Govil D, Graci C, Ha YR, Hurtado L, Kameda T, Lanspa M, Laursen CB, Lee F, Liu R, Meineri M, Montorfano M, Nazerian P, Nelson BP, Neskovic AN, Nogue R, Osman A, Pazeli J, Pereira-Junior E, Petrovic T, Pivetta E, Poelaert J, Price S, Prosen G, Rodriguez S, Rola P, Royse C, Chen YT, Wells M, Wong A, Xiaoting W, Zhen W, Arabi Y (2020). Multi-organ point-of-care ultrasound for COVID-19 (PoCUS4COVID): international expert consensus. Crit Care.

[12] Lichtenstein D (2017). Novel approaches to ultrasonography of the lung and pleural space: where are we now?. Breathe (Sheff).

[13] Bouhemad B, Mongodi S, Via G, Rouquette I (2015). Ultrasound for lung monitoring of ventilated patients. Anesthesiology.

[14] Boccatonda A, Cocco G, D’Ardes D, Delli Pizzi A, Vidili G, De Molo C, Vicari S, Serra C, Cipollone F, Schiavone C, Guagnano MT (2023). Infectious pneumonia and lung ultrasound: a review. J Clin Med.

[15] Bouhemad B, Brisson H, Le-Guen M, Arbelot C, Lu Q, Rouby JJ (2011). Bedside ultrasound assessment of positive end-expiratory pressure-induced lung recruitment. Am J Respir Crit Care Med.

[16] Tonelotto B, Pereira SM, Tucci MR, Vaz DF, Vieira JE, Malbouisson LM, Gay F, Simões CM, Carvalho Carmona MJ, Monsel A, Amato MB, Rouby JJ (2020). Costa Auler JO Jr. Intraoperative pulmonary hyperdistention estimated by transthoracic lung ultrasound: a pilot study. Anaesth Crit Care Pain Med.

[17] Nhat PTH, Van Hao N, Tho PV, Kerdegari H, Pisani L, Thu LNM, Phuong LT, Duong HTH, Thuy DB, McBride A, Xochicale M, Schultz MJ, Razavi R, King AP, Thwaites L, Van Vinh Chau N, Yacoub S (2023). VITAL Consortium; Gomez A. Clinical benefit of AI-assisted lung ultrasound in a resource-limited intensive care unit. Crit Care.

